# Protein lactylation: molecular mechanisms underlying lactate-driven tumorigenesis and cancer progression

**DOI:** 10.1080/15384047.2025.2603081

**Published:** 2025-12-18

**Authors:** Qianyun Xie, Lijuan Weng, Yuqing Hu, Qingsong Tao, Ruishuang Ma

**Affiliations:** a Department of Radiotherapy and Chemotherapy, The First Affiliated Hospital of Ningbo University, Ningbo, Zhejiang, China; b Ningbo University Health Science Center, Ningbo, Zhejiang, China

**Keywords:** Lactate, lactylation, cancer, epigenetic regulation, tumor microenvironment

## Abstract

Lactylation, a recently identified post-translational modification, has reshaped our understanding of lactate from a metabolic byproduct to a central regulator of tumor biology. Accumulating evidence reveals that lactate-driven lactylation orchestrates metabolic reprogramming, epigenetic remodeling, immune evasion, metastasis, and therapeutic resistance, thereby fueling malignant progression. Beyond histones, diverse non-histone substrates further expand its regulatory network across cancer signaling pathways. We highlight the crosstalk between lactylation and other modifications, its role in tumor heterogeneity, and the therapeutic opportunities arising from targeting this pathway. These insights establish lactylation as both a hallmark and a potential vulnerability of cancer, opening new avenues for precision oncology.

## 1 Introduction

Glycolysis involves the conversion of glucose into two pyruvate molecules, which are subsequently reduced to lactate by lactate dehydrogenase A (LDHA), a pivotal isoenzyme, in a process that concomitantly regenerates NAD⁺.^1^ Over the past decade, the perception of lactate has fundamentally shifted from that of a mere metabolic byproduct to a critical signaling molecule and regulator in tumor biology. Clinical studies have consistently reported profound dysregulation of lactate homeostasis within the tumor microenvironment (TME), characterized by markedly elevated lactate concentrations compared to adjacent normal tissues. This accumulation stems from a confluence of factors, including the altered metabolic phenotypes of tumor cells, the activation of hypoxia-inducible factor (HIF)-driven pathways, and complex interactions involving tumor-associated immune cells.^2^ Indeed, malignant tumors exhibit sustained activation of aerobic glycolysis, a phenomenon known as the Warburg effect, leading to excessive lactate production. Mounting evidence underscores lactate’s central involvement in diverse oncogenic processes, including metabolic reprogramming, protein lactylation, immune evasion, therapeutic resistance, epigenetic modulation, and tumor metastasis – all of which are strongly correlated with aggressive tumor phenotypes and unfavorable clinical outcomes in cancer patients.^3-5^ These findings provide a crucial framework for understanding the multifaceted mechanisms through which lactate promotes malignant progression and offer new avenues for therapeutic intervention.

### 1.1 Lactate: from metabolic byproduct to key signaling molecules in cancer

Lactate exists as two stereoisomers: L-lactate (S-configuration) and D-lactate (R-configuration). Although these enantiomers are mirror images in structure, they differ profoundly in biosynthetic routes and physiological functions. L-lactate is the central product of glycolysis and the Cori cycle, serving as both a major vehicle for energy transfer and the predominant isomer driving lactylation, thereby shaping cellular metabolism and survival.^6^ In contrast, D-lactate is generated only in small amounts through the methylglyoxal pathway, and its metabolic fate and physiological role have long remained underappreciated.^7^ In cancer metabolism, L-lactate sustains cell survival under glucose-limiting conditions by fueling mitochondrial oxidation via GLS1-dependent glutaminolysis.^8^ In contrast, D-lactate has recently emerged as a secreted metabolite of cancer cells that may contribute to tumor progression by rewiring the metabolism of surrounding normal cells.^9^ These divergent roles highlight the distinct contributions of lactate enantiomers to tumorigenesis and point to new opportunities for precision therapies targeting lactate metabolism. The recent development of genetically encoded biosensors (e.g., eLACCO2.1, R-iLACCO1, and BLac-6) enables isomer-specific detection and spatiotemporal imaging, offering powerful tools to dissect their differential functions in cancer.^10^^,^^11^

Lactate, once viewed primarily as a terminal metabolite of energy metabolism, is now recognized as a pleiotropic signaling molecule with profound biological significance, particularly within the tumor microenvironment (TME). Its signaling functions are multifaceted, encompassing several key mechanisms. First, lactate directly activates downstream signaling pathways via G protein-coupled receptors (GPCRs), notably GPR81 and GPR132. This engagement influences inflammation, TME remodeling, metabolic reprogramming, and cellular proliferation.^3^^,^^12-14^ Second, within the TME, lactate facilitates intercellular communication through ‘lactate shuttle’ mechanisms. For instance, lactate released by tumor cells can be internalized by cancer-associated fibroblasts (CAFs), supporting their metabolic demands and activating signaling pathways that establish a pro-tumorigenic feedback loop.^15^ Furthermore, lactate critically modulates immune responses, inhibiting dendritic cell maturation and promoting the differentiation of regulatory T cells (Treg), thereby fostering an immunosuppressive TME.^16^^,^^17^ Lactate also triggers pro-angiogenic signaling pathways, contributing to tumor vascularization and growth.^18^ Collectively, these findings have dramatically expanded the recognized functional repertoire of lactate, transforming its perception from a mere metabolic byproduct to a pivotal mediator of tumor progression.

A landmark discovery in 2019 by Zhang et al. identified histone lactylation as a novel post-translational modification (PTM), fundamentally reshaping our understanding of lactate’s biological roles.^19^ This breakthrough elegantly integrated metabolic reprogramming with epigenetic regulation, establishing a new paradigm within oncobiology.^5^^,^^19^ Specifically, lactate serves as a direct metabolic signal, modulating chromatin architecture via lysine lactylation of histones, thereby directly altering gene expression profiles. This histone lactylation alters chromatin accessibility by modifying the electrostatic charge of histones, consequently governing the transcriptional activity of key oncogenes (e.g., within the POM121-MYC-PD-L1 axis) and pivotal metabolism-associated genes such as HK1 and LDHA.^5^^,^^20^^,^^21^ The discovery of lactylation has thus unveiled crucial non-metabolic roles for lactate accumulated within the TME, elucidating how tumor cells can directly exploit metabolic intermediates to orchestrate gene expression. This position lactate as a critical molecular bridge linking oncogenic metabolic reprogramming to epigenetic dysregulation, thereby reinforcing its role as a key driver of malignant transformation and progression​​​​​progression​​​.

## 2 Lactylation: orchestrating metabolic and epigenetic reprogramming in cancer

Lactylation is a recently discovered post-translational modification (PTM) involving the covalent attachment of a lactyl group derived from lactate to the *ε*-amino group of protein lysine residues to form *ε*-N-lactyllysine.^19^^,^^20^^,^^22^ This modification can be broadly categorized based on its substrate into histone and non-histone protein lactylation. Histone lactylation, occurring at specific sites such as H3K18 and H3K9, directly alters the nucleosomal architecture to modulate chromatin accessibility and, consequently, gene transcription.^5^^,^^20^^,^^22^ In parallel, a growing body of evidence reveals that a diverse repertoire of non-histone proteins – including key metabolic enzymes, signaling mediators, and transcription factors –are also targets of lactylation.^23-26^ This finding indicates a pervasive regulatory role for this modification that extends far beyond chromatin dynamics. Thus, the identification of lactylation establishes a direct molecular bridge between cellular metabolism and protein function, offering unprecedented insight into how metabolic dysregulation can drive the pathogenesis and progression of diseases like cancer.^5^^,^^19^^,^^27^

**Figure 1.**
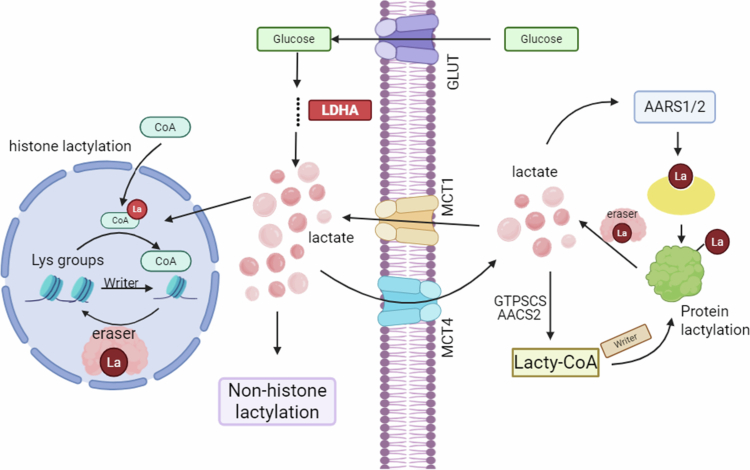
Overview of lactylation pathways in tumor cells. Tumor cells overexpress GLUT to uptake glucose, which is then reduced to lactate through glycolysis. Lactate transporters regulate lactate exchange between cells, with MCT1 mediating uptake and MCT4 being responsible for export. Protein lactylation can be categorized into histone lactylation and non-histone lactylation, involving the attachment of lactate to lysine residues in proteins to induce lactylation. This process involves direct lactate transfer via lactoyl-CoA-dependent or independent pathways involving AARS1/2. In the lactoyl-CoA-dependent pathway, lactate is converted to lactoyl-CoA (with GTPSCS and AACS2 potentially acting as lactoyl-CoA synthetases), after which writers transfer the lactoyl group to lysine residues on substrate proteins – a process reversible by erasers (created with biorender).

Two principal routes mediate the lactylation of proteins: an enzyme-catalyzed reaction dependent on lactyl-coenzyme A (lactyl-CoA), and a direct lactyl group transfer pathway catalyzed by aminoacyl-tRNA synthetases (AARSs). The lactyl-CoA-dependent mechanism shares striking similarities with other acylation processes, such as acetylation and butyrylation. Within this pathway, lactyl-CoA directly donates the lactyl moiety, which is then covalently attached to lysine residues on target proteins via the catalytic activity of p300, a well-established histone acetyltransferase. Interestingly, p300 exhibits a high binding affinity for lactyl-CoA, thus supporting the enzyme’s catalytic efficiency for lactyl group transfer.^28^ Succinyl-CoA synthetase (GTPSCS) and acetyl-CoA synthetase 2 (ACSS2) are among the enzymes implicated as potential lactyl-CoA synthases, contributing to intracellular pools of this essential intermediate.^29^^,^^30^ Moreover, beyond p300-mediated lactylation, members of the AARS family – notably AARS1 and AARS2 – have been identified as dual-function proteins capable of sensing L-lactate and acting as lactyltransferases (Figure 1). These enzymes are critically involved in mediating global protein lactylation in response to elevated intracellular L-lactate concentrations, thereby integrating metabolic signals with post-translational regulatory networks.^31-33^ Both AARS1/2 bind L-lactate with micromolar affinity and, in an ATP-dependent manner, catalyze the formation of lactyl-AMP, which is subsequently transferred to lysine residues on substrate proteins.^33^ In tumor cells, AARS1 translates fluctuations in lactate levels into widespread proteomic remodeling, including lactylation of key effectors such as p53, cGAS, and BLM.^34-36^ For example, AARS1-mediated lactylation of p53 at K120 and K139 within its DNA-binding domain disrupts liquid–liquid phase separation, DNA interaction, and transcriptional activity – an alteration strongly correlated with poor prognosis in cancers harboring wild-type p53.^34^ Under chemotherapeutic stress, AARS1 further drives hyperlactylation of BLM at K24, which stabilizes BLM by blocking MIB1-mediated ubiquitination, thereby enhancing homologous recombination repair and promoting drug resistance.^36^ Notably, AARS2 specifically catalyzes cGAS lactylation, dampening its activity and modulating innate immune responses.^37^ Together, these findings position AARS1/2 as metabolic–epigenetic hubs that couple tumor metabolic reprogramming with proteome-wide regulation during cancer progression.^33^^,^^34^

### 2.1 Lactylation-driven positive feedback loops fuel glycolysis in cancer

Metabolic reprogramming in tumor cells often involves a preferential upregulation of glycolytic pathways, leading to elevated lactate levels within the tumor microenvironment (TME). This accumulation of lactate, in turn, promotes protein lactylation, thereby reinforcing tumor-specific metabolic phenotypes and establishing a positive feedback loop between glycolysis and lactylation to accelerate tumor progression. Indeed, enolase 1 (ENO1) and pyruvate kinase M2 isoform (PKM2) – two key glycolytic enzymes – have been identified as critical lactylation targets. Within the TME, lactylation primarily exerts positive regulatory effects on these targets. For example, lactylation at lysine 62 (K62) on PKM2 enhances its tetramer stability and kinase activity, promoting pyruvate production while concurrently diminishing its nuclear translocation. This modification attenuates pro-inflammatory macrophage-mediated inhibition of glycolysis, thereby preserving the metabolic advantage of tumor cells.^38^ Similarly, lactylation at lysine 343 (K343) on ENO1 has been shown to decrease its thermal stability, potentially increasing its catalytic efficiency and further augmenting glycolytic flux.^39^ Disruption of this delicate equilibrium favors the establishment of a lactylation-mediated positive feedback circuit, further accelerating tumor growth.

Lactate can also directly modulate the expression of genes involved in glycolysis through histone lactylation. For instance, in pancreatic ductal adenocarcinoma (PDAC), a microenvironment rich in lactate triggers lactylation at histone H3 lysine 18 (H3K18la), leading to a significant upregulation of kinases such as TTK and BUB1B. TTK, in turn, phosphorylates and activates lactate dehydrogenase A (LDHA), thereby increasing lactate production, while BUB1B increases the expression of the histone acetyltransferase p300. This amplifies global lactylation levels and establishes a self-reinforcing “glycolysis-H3K18la-TTK/BUB1B” positive feedback loop.^40^ A comparable mechanism has been observed in colorectal cancer, exemplified by the NSUN2/YBX1/m5C-*p* regulatory axis. Here, lactylated ENO1 promotes glycolysis and lactate production, activating NSUN2 transcription via histone modifications, thereby forming a self-sustaining metabolic reprogramming network.^41^

### 2.2 Mitochondrial dysfunction and lactylation: a metabolic tug-of-war

Tumor cells exhibit marked morphological changes in mitochondria, transitioning from highly organized reticular networks to fragmented, punctate structures, often with accompanying mitochondrial hyperpolarization and suppressed oxidative phosphorylation (OXPHOS).^42^ Although OXPHOS maintains the capacity for glucose oxidation in cancer cells,^43^ lactylation may modulate this process.^44^ Indeed, knockdown of methyltransferase-like 15 (METTL15) – a mitochondrial rRNA methyltransferase essential for ribosomal assembly and OXPHOS activity – has been shown to increase histone H4K12 and H3K9 lactylation levels while simultaneously downregulating the expression of mitochondrially-encoded proteins. This leads to OXPHOS dysfunction and elevated reactive oxygen species (ROS), indicating a possible link between lactylation and mitochondrial impairment.^44^^,^^45^ Protein lactylation plays a critical role in regulating mitochondrial respiratory chain function. Mechanistically, under hypoxic conditions, AARS2 can catalyze lactylation at lysine residues K336 of the pyruvate dehydrogenase complex subunit PDHA1 and K457/458 of carnitine palmitoyltransferase 2 (CPT2) – both resident mitochondrial proteins. Conversely, SIRT3 reverses these lactylation modifications on PDHA1 and CPT2, thereby restoring OXPHOS activity,^46^ impacting mitochondrial quality control. The Numb/Parkin pathway, a key mediator of mitochondrial quality control, can influence tumor cell fate by metabolically regulating histone lactylation. For example, in prostate and lung adenocarcinoma, mitochondrial dysfunction causes defects in the Numb/Parkin pathway, triggering metabolic reprogramming and increased lactate production. This, in turn, upregulates neuroendocrine-related gene expression through histone lactylation, ultimately driving acquired drug resistance.^47^ Consistent with this, hypoxia-induced glycolytic lactate can enter mitochondria, where lactylation of mitochondrial enzymes such as PDHA1 at K336 and CPT2 at K457/458 inhibits their activities, limiting both OXPHOS and ROS production.^46^

Beyond these effects, accumulating evidence demonstrates that lactylation acts through two principal modes: one is by directly modifying the subunits of respiratory chain complexes to change their enzyme activity. For example, lactylation at the K531 site of the ATP5F1A protein damages ATP synthase activity, leading to increased reactive oxygen species (ROS) production, reduced ATP yield, and triggering abnormal mitochondrial structure.^48^ The other mechanism involves indirectly affecting respiratory chain function through the regulation of metabolic pathways such as NADPH generation (such as the IDH1-dependent pathway). In renal cell carcinoma (RCC), lactylation modifies proteins such as mitochondrial malate dehydrogenase 2 (MDH2) to reprogram mitochondrial metabolism to increase oxidative stress resistance and promote tumor progression.^49^ These findings reveal that lactylation, as a metabolic signal integration mechanism, can convert intracellular hypoxia and lactate level changes into precise regulation of mitochondrial respiratory chain function.^46^ The regulation of the mitochondrial respiratory chain by lactylation also manifests at the structural level. For example, lactylation of the Mic60 protein can affect the remodeling and metabolic activation of mitochondrial cristae, which may be achieved by changing the density and compactness of the cristae.^50^ Lactylation can also promote mitochondrial fragmentation and dysfunction, as observed with Fis1 K20 lactylation, which drives excessive fission, and ALDH2 K52 lactylation, which exacerbates tubular injury and mitochondrial impairment.^51^^,^^52^ Collectively, these findings reveal lactylation as a signaling axis that integrates hypoxia and lactate accumulation into the precise regulation of mitochondrial respiration, encompassing OXPHOS activity, metabolic reprogramming, cristae remodeling, and organelle dynamics. Such insights provide new perspectives on disease mechanisms and potential therapeutic targets for mitochondrial dysfunction.

## 3 Lactylation’s multifaceted role in tumorigenesis

### 3.1 Lactylation as a driver of oncogene activation

Epigenetic alterations, including DNA methylation and histone modifications, are well-established mechanisms by which tumor suppressor genes can be silenced or oncogenes activated, thereby contributing to malignant transformation.^53^^,^^54^ Increasingly, lactylation is emerging as a central epigenetic regulator, modulating the expression of key cancer-related genes and plays a pivotal role in tumor initiation. For example, in hepatocarcinogenesis, histone lactylation marks such as H3K9la and H3K56la promote tumor development via a dual mechanism: they increase the self-renewal capacity of liver cancer stem cells (LCSCs), as evidenced by upregulated CD133 expression, and concurrently accelerate cell cycle progression through increased expression of Cyclin D1 and Cyclin E1. Notably, genetic ablation of these lactylation sites significantly reduces the tumorigenic potential of LCSCs, highlighting the direct regulatory function of histone lactylation in tumor initiation.^55^ Similarly, in breast cancer, increased lactate metabolism elevates global histone lactylation levels, driving overexpression of the oncogene c-Myc, which, in turn, upregulates the splicing factor serine/arginine-rich splicing factor (SRSF10). SRSF10 orchestrates aberrant splicing of MDM4 and Bcl-x transcripts, ultimately accelerating tumor progression.^56^ Furthermore, histone lactylation has been shown to activate the transcription of platelet-derived growth factor receptor *β* (PDGFRβ) in clear cell renal cell carcinoma (ccRCC), promoting disease progression;^57^ targeting histone lactylation effectively suppresses ccRCC cell proliferation in vivo. Likewise, in cervical cancer, lactylation enhances the expression of the oncogenic protein DCBLD1 (the discoidin, CUB, and LCCL domain-containing), which activates the pentose phosphate pathway, thereby amplifying proliferative signaling within tumor cells.^58^ Taken together, these findings underscore the importance of lactylation as a key epigenetic modification that drives oncogene activation in diverse cancers, suggesting its potential as a therapeutic target for early cancer intervention.

### 3.2 Lactylation-mediated disruption of tumor suppressor pathways

Lactylation directly modifies key molecules within various tumor suppressor pathways, thereby promoting tumorigenesis. In the p53 pathway, for instance, p53-a critical regulator of genomic stability, undergoes lactylation at lysine residues K120 and K139. This modification induces conformational changes in its DNA-binding domain, significantly impairing p53’s ability to bind to target gene promoters. Consistent with these findings, research by Zhou et al. demonstrated a strong inverse correlation between p53 pathway activity and lactylation levels in lactate-rich breast cancer tissues. In vitro experiments further confirmed that treatment with sodium lactate selectively increases p53 lactylation without affecting total p53 protein expression.^34^ Beyond p53, PTEN (phosphatase and tensin homolog), the first identified tumor suppressor with phosphatase activity, functions as a direct inhibitor of the PI3K/AKT signaling pathway.^59^ In prostate cancer, PTEN loss leads to aberrant PI3K/AKT activation, forming a malignant feedback loop in conjunction with lactylation. Specifically, lactylation promotes this cycle by activating the Wnt/β-catenin pathway to increase glycolysis and shape an immunosuppressive microenvironment. Moreover, lactylation at histone H3 lysine 18 (H3K18la) upregulates PD-L1 expression, suppressing CD8+ T cell cytotoxic function.^60^^,^^61^ Recent studies have indicated that lactylation also disrupts the Hippo signaling pathway by modulating the activity of the YAP/TAZ proteins, thereby relieving their inhibitory control over cell proliferation. This mechanism is evident in the circXRN2-Hippo regulatory axis identified in bladder cancer.^62^^,^^63^ Collectively, these findings reveal a complex regulatory network whereby lactylation influences tumor initiation through interconnected metabolic reprogramming and epigenetic modulation, highlighting its central role in the dysregulation of tumor suppressor pathways.^64^^,^^65^

## 4 Lactylation enhances tumor metastasis and invasion

### 4.1 Lactylation’s impact on EMT-related genes

Emerging evidence indicates that lactylation plays a critical role in tumor invasion and metastasis, largely through the regulation of epithelial‒mesenchymal transition (EMT)-related genes. Specifically, lactylation of histone H3 at lysine 18 (H3K18la) significantly upregulates the transcription of vascular cell adhesion molecule 1 (VCAM1), which subsequently activates the AKT-mTOR signaling pathway; this, in turn, promotes uncontrolled tumor cell growth, stimulates EMT initiation and progression, and accelerates metastatic spread.^66^ At the molecular level, RHOF mediates the nuclear translocation and lactylation of Snail1, dramatically increasing the migration and invasiveness of tumor cells. Importantly, genetic knockout of Snail1 abrogates the pro-EMT effects of lactate, highlighting the pivotal role of Snail1 lactylation in this process.^67^ Together, these studies demonstrate that lactylation directly modulates the molecular pathways of core EMT transcription factors via epigenetic mechanisms.

### 4.2 Lactylation-mediated angiogenesis

Lactylation also exerts a regulatory influence on tumor angiogenesis. Mechanistically, activation of the VEGF signaling pathway significantly induces lactylation of histone H3 at lysine 9 (H3K9la) in endothelial cells. This epigenetic modification selectively binds to the promoter regions of pro-angiogenic genes, markedly enhancing their transcriptional activity. Concurrently, the expression of the de-lactylase HDAC2 is downregulated, establishing a self-reinforcing positive feedback loop. Importantly, targeting glycolytic metabolism to reduce H3K9 lactylation levels effectively inhibits tumor neovascularization.^68^ In prostate cancer, the expression of the cell migration-inducing protein KIAA1199 is positively correlated with HIF-1α activity and the extent of angiogenesis. HIF-1α-mediated histone lactylation further upregulates KIAA1199 expression, thereby enhancing the angiogenic capacity of tumor tissues (Figure 3). Knockdown of KIAA1199 disrupts hyaluronic acid (HA)-dependent VEGFA signaling, ultimately suppressing angiogenesis in prostate cancer.^69^ Furthermore, in microglial cells, the lactylation writer enzyme p300 promotes histone lactylation to upregulate YY1 expression, which activates the fibroblast growth factor (FGF) signaling pathway and promotes angiogenesis.^70^ These findings demonstrate that lactylation orchestrates multiple pathways to synergistically regulate the VEGF signaling network, establishing a complex pro-angiogenic mechanism within the tumor microenvironment.

### 4.3 Lactylation contributes to immunosuppression of TME

Lactate has emerged as a key regulator of immune response, as various immune cells in the lactate-rich environment tend to exhibit an immunosuppressive feature in cancers.^71-73^ The underlying mechanism by which lactate acts still cannot be fully explained despite the lactate receptor GPR81 signaling.^74^^,^^75^ Emerging evidence shows that epigenetic regulation arises as a critical lactate-driven mechanism to shape immune adaption. In 2019, lactylation of specific histone residues (H3K18) was first recognized in septic macrophages which promotes cellular homeostasis towards M2 polarization and is associated with a macrophage-resolving phenotype.^19^ Recently, Veglia et al. further demonstrated that glycolytic monocyte-derived macrophages (MDMs) are major contributors to the immunosuppression associated with tumor-associated macrophages (TAMs) and intracellular lactate upregulates IL-10 expression in TAMs via histone lactylation in glioblastoma.^76^ In a mouse model of prostate cancer, phosphatidylinositol 3-kinase (PI3K) inhibition was reported to diminish tumor-derived lactate and lactylation within TAMs, thereby improving their phagocytic capacity.^60^ The molecular regulation of lactate is potentially to favor the tetrameric form of PKM2 through lactylation of K62 in macrophages, inducing anti-inflammatory effects including reduced migration and pro-inflammatory cytokine release.^38^ Additionally, lactate induces K63 lactylation of Endosulfine alpha, a crucial step that triggers STAT3-CCL2 signaling, and the elevated CCL2 secreted by tumor cells facilitates TAM recruitment to the TME.^77^

The histone lactylation in malignant cells facilitates CD8+ T cell dysfunction and immune evasion. In head and neck squamous cell carcinoma, Wang et al. showed that lactate accumulation promotes H3K9 lactylation and downstream IL-11 activation, leading to exhaustion of CD8+ T cells by upregulating immune checkpoints through the JAK2/STAT3 signaling pathway.^78^ In addition, lactate-upregulated H3K18la directly binds to the B7-H3 promoter in conjunction with the transcription factor Creb1 and its co-activator Ep300, leading to increased B7-H3 expression and contributing to tumor progression by compromising the proportion and cytotoxicity of tumor-infiltrating CD8+ T cells.^79^ In contrast, interruption of glycolysis via LDHA knockdown or treatment with sodium oxamate restored the cytotoxicity of CD8+ T cells, effectively augmenting the immunotherapy response.^79^^,^^80^

Lactate exerts a profound impact on immunetherapy efficacy not only by directly impairing CD8+ T cell killing activity but also by enhancing the function of regulatory T (Treg) cells. Gu et al. found that lactate regulates Treg cells through lactylation of MOESIN at the Lys72 residue, which enhances TGF-*β* signaling in the TME.^81^ Similar to Treg cells, FOXP3+ NKT-like cells also had highly activated glycolysis pathway and pyruvate metabolism, accompanied with immunosuppressive features and hyperlactylation.^82^ Futhermore, Th17 cells, in the presence of lactate, undergo epigenetic modification through histone H3K18 lactylation, reprogramming them into Treg cells through IL-2-driven decreased IL-17 production and increased Foxp3 expression.^83^

However, new perspectives were proposed that lactate and lactylation act as dual regulators of T-cell-mediated tumor immunity in a context-dependent manner.^84^ A recent study in Nature Immunology elaborated that the enrichment of H3K18la and H3K9la acts as transcription initiators of key genes regulating CD8+ T cell function.^85^ H3K9la is enriched in both naive and activated states, while H3K18la is specific to activated CD8+ T cells. Further, H3K18la and H3K9la marks were not enriched in exhaustion-associated genes, whereas the enrichment of H3K9ac in exhaustion-associated genes in exhausted CD8+ T cells generated. Meanwhile, the GSEA analysis showed that H3K18la is associated with inflammatory responses and glycolytic metabolic pathways, while H3K9la is closely related to oxidative phosphorylation, mitochondrial respiration, and fatty acid metabolic pathways. These results not only emphasize the crucial role of H3K18la and H3K9la in regulating T cell mitochondrial dynamics and energy metabolism, but also reveal their differential regulation of gene expression under different T cell states.

**Figure 2.**
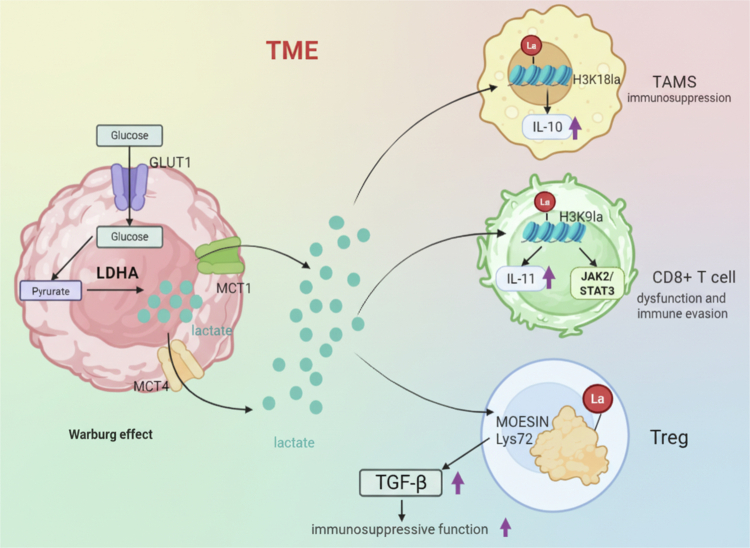
Lactate-driven lactylation shapes the immunosuppressive TME. Excess lactate produced by tumor glycolysis reprograms immune cells through distinct lactylation events, enhancing TAM-mediated immunosuppression, driving CD8⁺ T-cell exhaustion via IL-11/JAK2-STAT3 signaling, and reinforcing Treg suppressive function (created with biorender).

Histone lactylation also has inhibotory effects on myeloid cells. Tumor-infiltrating myeloid cells (TIMs) serve as key effector cells mediating immune escape, and their functional status is significantly regulated by lactylation modification. Research has found that H3K18la modification can promote the transcriptional activation of the methyltransferase METTL3. Meanwhile, lactylation modification directly enhances the binding ability of METTL3 protein to bind to RNA m6A-modified targets, and this dual regulatory mechanism ultimately promotes TIM-mediated immunosuppressive effects.^86^ Neutrophils, the most abundant immune cells in peripheral blood, have long been regarded as the body's first line of defense against infections, but recent studies have gradually revealed their critical role in cancer progression. Ugolini et al. recently found that a subpopulation of neutrophils highly expressing CD71 (transferrin receptor) is the main executor of immune suppression.^87^ These types of cells preferentially aggregate in the hypoxic/high glycolytic regions of tumors, and hypoxia drives high lactate accumulation and histone lactylation, leading to immunosuppressive functions of CD71+ neutrophils through ARG1. Targeting histone lactylation with the anti-epileptic drug isosafrole blocked CD71+ neutrophil immunosuppressive ability, delayed tumor progression and sensitized brain tumors to immunotherapy (Figure 2).

## 5 Lactylation drives therapeutic resistance in cancer

### 5.1 Lactylation: a key obstacle to chemotherapy efficacy

The abnormal accumulation of lactate within the tumor microenvironment is increasingly recognized as a critical factor in establishing chemotherapy resistance. Lactylation, an epigenetic modification influenced by this metabolic milieu, has been shown to reduce tumor cell sensitivity to various chemotherapeutic agents through dual regulation of metabolic pathways and DNA damage repair systems.

In colorectal cancer stem cells, lactylation of histone H4 at lysine 12 (H4K12la) transcriptionally activates glutamate‒cysteine ligase catalytic subunit (GCLC) expression, significantly inhibiting ferroptosis pathways. This leads to pronounced resistance to chemotherapy drugs both in vitro and in vivo. Conversely, knockdown of p300 or LDHA, which reduces lactylation levels, markedly enhances tumor cell sensitivity to chemotherapy.^88^ Chen et al. further demonstrated that lactylation enhances the homologous recombination repair activity of the MRE11 protein, substantially increasing tumor cell tolerance to cisplatin and PARP inhibitors.^89^^,^^84^ Importantly, penetrating peptides targeting lactylation sites of MRE11 effectively reverse this resistant phenotype, suggesting that co-targeting ferroptosis suppression and enhanced DNA repair pathways may provide a novel approach to overcome chemoresistance. Preclinical studies have confirmed the therapeutic potential of targeting lactylation regulatory networks – for example, by inhibiting lactate dehydrogenase activity or employing specific small molecule inhibitors – to reverse drug resistance in various tumor models, including mouse xenografts and organoid cultures.^88^^,^^89^ In bladder cancer, Li et al. demonstrated that H3K18la modification activates the key transcription factors YBX1 and YY1, promoting cisplatin resistance. Specific inhibition of H3K18la significantly restores cisplatin sensitivity in resistant bladder cancer cells, presenting a promising clinical target to overcome drug resistance.^19^ Moreover, lactylation at lysine 388 (K388) of the NBS1 protein positively correlates with clinical platinum drug resistance and serves as an independent predictor of poor prognosis, suggesting that targeting lactate metabolism pathways or selectively inhibiting lactylation may improve chemotherapy outcomes.^90^ Finally, in colorectal cancer, tumor-derived lactate induces H3K18la modification, upregulating the autophagy regulator RUBCNL and thereby enhancing resistance to bevacizumab treatment.^91^ In colorectal cancer, excessive lactylation of XLF impairs non-homologous end joining repair, thereby contributing to chemotherapy resistance.^92^

### 5.2 Lactylation-mediated DNA damage repair in radiotherapy resistance

Lactylation emerges as a critical regulator of DNA damage repair (DDR) processes that contribute to radiotherapy resistance in multiple cancers. Li et al. identified a mechanism in glioblastoma in which the ALDH1A3-PKM2-lactate-XRCC1 axis promotes therapeutic resistance by enhancing DNA damage repair. Notably, targeting this pathway significantly increased tumor sensitivity to radiotherapy,^93^ highlighting its potential as a therapeutic vulnerability.

**Figure 3.**
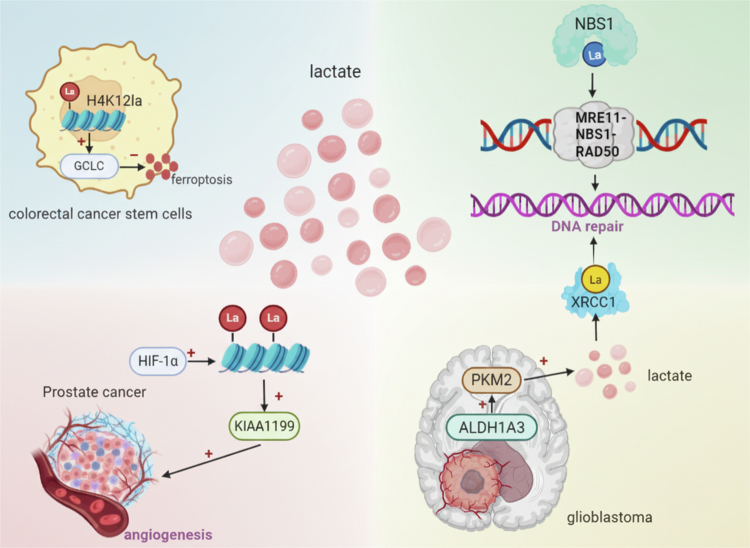
Lactylation promotes therapeutic resistance and angiogenesis. In the lactate-rich TME, histone and non-histone lactylation support ferroptosis evasion, increase DNA damage repair and radioresistance, and upregulate pro-angiogenic factors, collectively driving tumor progression and treatment resistance (created with biorender).

Lactylation modulates radiotherapy sensitivity via diverse pathways within the DDR system. For instance, NONO protein, through liquid‒liquid phase separation (LLPS), recruits nuclear EGFR and the DNA‒PK complex, substantially improving the efficiency of non-homologous end joining (NHEJ) repair and consequently promoting radiotherapy resistance. This mechanism correlates with poor prognosis in several solid tumors.^94^^,^^95^ At the molecular level, lactate-induced lactylation of NBS1 protein at lysine 388 (K388) stabilizes the MRN complex structure, facilitating the precise recruitment of homologous recombination repair proteins to DNA double-strand break sites, thereby enhancing radiotherapy resistance. TIP60 acetyltransferase has been identified as a key lactylation “writer” enzyme in this process.^90^ Further, in glioblastoma, ALDH1A3 allosterically activates PKM2, which reprograms tumor metabolism, leading to lactate accumulation. This, in turn, induces lactylation of XRCC1 at lysine 247 (K247), promoting nuclear translocation of XRCC1, enhancing DNA repair, and ultimately driving resistance against radiochemotherapy^93^ (Figure 3).

In summary, lactylation significantly promotes therapeutic resistance in cancer by regulating diverse processes, such as ferroptosis, DNA damage repair, and autophagy. To provide a clearer overview of the tissue-specific roles and therapeutic implications of lactylation, we have summarized representative lactylation targets, functional consequences, and preclinical evidence in Tables 1 and 2 below.

**Table 1.** Comparative examples of tissue-specific lactylation.

Cancer type	Lactylation target/mechanism	Phenotype	Therapeutic implication	Refs
Glioblastoma	XRCC1 K247 lactylation via ALDH1A3→PKM2→lactate	Enhanced DDR, radioresistance	Inhibit ALDH1A3/PKM2 axis or block XRCC1 lactylation	[93]
Colorectal cancer	H4K12la ↑ GCLC → ferroptosis inhibition	Chemoresistance	Inhibit p300/LDHA to reduce H4K12la; combine ferroptosis inducers	[88]
Colorectal cancer	H3K18la ↑ RUBCNL (autophagy enhancer)	Chemoresistance	Target lactate/H3K18la or autophagy axis	[91]
Multiple solid tumors	NBS1 K388 lactylation stabilizes MRN complex	Chemo-tolerance	Target TP60-dependent writing or NBS1 K388la	[90]
Bladder cancer	H3K18la → YBX1/YY1 transcriptional program	Cisplatin resistance	Pharmacologic inhibition of H3K18la	[19]

**Table 2.** Representative histone/non-histone lactylation targets, functions, and contexts.

Target	Type	Function/Pathway	Cancer type/model
H3K18la	Histone	Immune evasion; EMT genes (e.g., VCAM1)	NSCLC/gastric/CRC
H3K9la	Histone	Pro-angiogenic transcription in ECs	Endothelium/pan-tumor
H4K12la	Histone	↑GCLC; ferroptosis suppression	CRC stem cells
PKM2 K62	Non-histone	Stabilizes tetramer; glycolysis tuning	Macrophages/tumor TME
ENO1 K343	Non-histone	↑Glycolytic flux	CRC
PDHA1 K336	Mitochondrial enzyme	Limits pyruvate dehydrogenase activity	Hypoxic tumors
CPT2 K457/K458	Mitochondrial enzyme	Restricts FAO and OXPHOS	Hypoxic tumors
DCBLD1	Non-histone scaffold	PPP activation	Cervical cancer
NBS1 K388	DDR factor	Stabilizes MRN, HR/NHEJ efficiency	Multiple tumors
XRCC1 K247	DDR factor	↑NHEJ/BER coordination	Glioblastoma
PDGFRβ	RTK	↑Proliferation signaling	ccRCC
c-Myc	TF	Splicing rewiring (SRSF10)	Breast cancer

## 6 Conclusion and future directions

The discovery of lactylation modification has revealed a close connection between metabolic abnormalities and epigenetic regulation in tumors. It plays a significant role in tumor development by regulating gene transcription, controlling metabolic processes, and affecting the immune microenvironment. Although preliminary studies have elucidated the mechanisms by which lactylation initiates, invades, metastasizes, evades the immune system, and develops resistance to treatments, there are still several fundamental gaps that need to be addressed.

First, the interaction network between lactylation and other post-translational modifications remains to be systematically parsed. An increase in lactylation levels competitively inhibits acetylation at the same lysine site and shares p300 as a catalyst, thereby dynamically regulating gene transcription. This competitive relationship arises from a metabolic shunting of the glycolysis pathway: when pyruvate tends to be converted into lactate rather than acetyl-CoA, lactylation modifications dominate, while acetylation levels decrease accordingly. This metabolic “switch” directly influences the tendency of cellular fate toward malignant transformation.^96^ Additionally, lactylation exhibits synergistic or antagonistic interactions with phosphorylation, ubiquitination, and other modifications. For example, the stability and function of ANXA2 are simultaneously regulated by lactylation, acetylation, ubiquitination, and phosphorylation, with these modifications collectively maintaining the stemness and radiotherapy resistance of tumor stem cells (GSCs).^97^ The cross-regulation between lactylation and phosphorylation is also evident in the positive feedback loop of ROS: H3K18 lactylation activates the transcription of the duox gene, promoting ROS production, and in turn, ROS further increase the level of H3K18 lactylation. This loop is also regulated by the phosphorylation signaling pathway.^98^ Lactylation and ubiquitination have synergistic effects on regulating protein stability and drug resistance. Lactylation upregulates the expression of ubiquitination-related enzymes (such as NEDD4, HECTD2), promoting the ubiquitination and degradation of key proteins (such as PTEN, KEAP1), leading to chemoresistance.^99^^,^^100^ Lactylation and crotonylation are both forms of lysine acetylation modifications.^101^ Although their regulatory enzyme systems partially overlap (such as p300,CBP, HBO1 as common writers, HDAC1-3, SIRT1-3 as common erasers),^32^^,^^102^ they each have unique recognition and regulatory mechanisms.^103^^,^^104^ Both participate in gene expression regulation and intracellular signaling pathways but may function through different molecular mechanisms.^105^ For example, histone H3 lysine 27 crotonylation (H3K27cr) guides gene transcription repression rather than activation,^106^ while lactylation is associated mainly with transcription activation.^32^ Lactolysis involves cross-regulation in the tumor microenvironment, where enhanced glycolytic metabolism leads to lactate accumulation and histone lactylation, which may also affect the level of crotonylation.^21^^,^^107^

Secondly, the enzymatic mechanism is unclear. The specific recognition mechanism of lactylation enzymes for substrates is unknown, especially the selective discrimination mechanism between acetylation modifications. In the future, structural biology (such as AlphaFold, X-ray crystallography) can be used to analyze the structure of enzyme-substrate complexes to predict dynamic conformational changes in the binding domain, elucidate the recognition mechanism, and thus provide a foundation for developing highly selective drugs.^108^ The identification of lactylation “eraser” is significantly lagging, severely constraining the analysis of the dynamic balance of modifications. Current research has only partially clarified the functions of lactyltransferases (such as KAT2A), while specific de-lactylating enzymes have yet to be discovered, leading to an unclear mechanism of precise regulation of the pathway under lactate fluctuations.^109^ In the future, structural biology needs to be combined to analyze the catalytic active center of enzymes, and CRISPR-Cas9 technology can be used to screen candidate eraser enzymes (such as SIRT, HDAC family) in patient-derived organoids (PDOs) to reveal the plastic regulatory mechanisms of lactylation under metabolic stress.

Third, the spatiotemporal dynamics of lactylation and the lack of clinical translation tools constitute a double bottleneck. The real-time monitoring and tissue-specific delivery systems for lactylation dynamics are key challenges that need to be addressed. The real-time process of how lactylation responds to metabolic fluctuations in the tumor microenvironment (such as hypoxia or nutrient deprivation) and drives epigenetic reprogramming has not yet been visualized.^64^ The levels of lactylation in the tumor microenvironment fluctuate with changes in metabolic status, so the development of advanced technologies such as nanotechnology will be crucial. Nanosensors can achieve real-time monitoring of spatiotemporal lactylation dynamics in complex tumor microenvironments.^110^ At the same time, the lack of highly selective inhibitors and non-invasive biomarker detection methods severely hinders clinical translation.^100^ Achieving precise delivery to specific tumor tissues is another key challenge. Nanocarriers provide promising platform for the targeted delivery of novel lactylation inhibitors or combination therapy drugs, enhancing tumor specificity, overcoming biological barriers, and minimizing systemic toxicity. Existing studies have shown that inhibitors targeting lactylation enzymes such as GCN5 or specific lactylation sites (such as XLF K288) have therapeutic potential,^92^ but these interventions need to overcome barriers in the tumor microenvironment while avoiding off-target effects in normal tissues. Additionally, lactylation is closely related to multiple mechanisms such as tumor metabolic reprogramming and immune escape, which requires the delivery system to be able to regulate multiple pathways synergistically.^16^ The current understanding of “writer”, “eraser”, and “reader” of lactylation is still incomplete,^100^ which also limits the rational design of targeted delivery systems. Future efforts need to develop multi-omics integration methods to construct a dynamic, multi-layered “lactylation modification-tumor microenvironment” interaction network model. This model not only elucidates the spatiotemporal distribution patterns of lactylation modifications and their effects on the tumor microenvironment but also identifies new targets for precision medicine.^65^ At the same time, the long-term physiological effects of lactylation interventions need to be comprehensively evaluated, especially in the context of their synergistic or antagonistic effects when combined with immunotherapy or radiation therapy.^64^ Solving these challenges will promote the transition of lactylation from basic research to clinical applications, providing new breakthroughs for tumor treatment. It is suggested to develop technologies capable of real-time, in-situ visualization of lactylation dynamic processes (such as novel biosensors and imaging techniques) and to design and develop high-selectivity small molecule inhibitors. Utilizing nanocarrier technologies, lactylation-regulating drugs can be specifically delivered to tumors to protect normal tissues.

Finally, current research on the interaction between lactylation and RNA is still in its early stages. Although studies have suggested that lactylation can regulate RNA modifications such as m⁶A, m⁵C, and m¹A by affecting the activity or expression of RNA modification enzymes (such as METTL3, METTL16), thereby influencing mRNA stability, translation efficiency, and immune function, the specific mechanisms involved remain unclear. For example, whether lactylation directly modifies RNA-binding proteins (such as the YTHDF family, IGF2BP3) and how these modifications affect their binding ability and function with RNA still require in-depth exploration. It is necessary to develop lactylation-related biomarkers based on RNA. By integrating multi-omics data (such as single-cell transcriptomics, spatial transcriptomics, and lactylationomics), lactylation-related RNA signatures can be constructed to predict treatment responses and patient prognosis. Machine learning models can be used to identify key RNA molecules (such as m6A modification enzymes, lactate metabolism enzyme transcripts, and immune-related ncRNAs) and evaluate their associations with lactylation levels, immune cell infiltration, and treatment resistance. Additionally, the role of lactylation in regulating the expression and function of non-coding RNAs (ncRNAs) such as circRNAs and lncRNAs has been almost unexplored, especially in the tumor microenvironment, where lactylation influences immune responses and tumor progression through RNA metabolism reprogramming, which still needs systematic elucidation. In the future, it will be necessary to elucidate whether lactylation affects the biogenesis, stability, or function of ncRNAs, and whether the feedback of these ncRNAs regulates the expression or activity of lactylation-related enzymes (such as LDHA, HDACs). Advanced technical platforms are also needed to promote mechanistic and translational research. Spatial multi-omics technologies (such as the combination of spatial transcriptomics and metabolic imaging) can reveal the spatial and temporal heterogeneity of lactylation and RNA expression; CRISPR screening can identify key genes regulating the cross-talk between lactylation and RNA; and nanoparticle delivery systems can achieve co-delivery of RNA drugs and lactylation inhibitors, improving targeting and efficacy.^111^

In summary, breakthroughs in lactylation research require a multidisciplinary approach: from the analysis of enzymatic mechanisms to decoding immune microenvironment heterogeneity, from the development of spatiotemporal dynamic tracking technologies to the innovation of clinical translation tools. The future should focus on integrating basic mechanisms with clinical applications and targeting the dynamic balance of lactylation to reshape the tumor immune microenvironment, ultimately promoting the development of precise treatment strategies.
